# The New Frontiers of Artificial Organ Engineering

**DOI:** 10.3390/bioengineering13060637

**Published:** 2026-05-29

**Authors:** Vincenzo Piemonte, Mario Merone

**Affiliations:** Department of Engineering, University Campus Bio-Medico di Roma, Via Alvaro del Portillo, 21, 00128 Rome, Italy

Artificial organ engineering is entering a new phase of maturity. What was once primarily a field devoted to functional substitution is now evolving into a multidimensional discipline that integrates biomaterials science, tissue engineering, computational modeling, artificial intelligence, additive manufacturing, and translational medicine. This transformation is redefining both the aims and the methods of the field. For these reasons, artificial organs are no longer conceived solely as inert replacements for failing biological functions, but they are being designed as dynamic, adaptive, and patient-centered systems capable of supporting regeneration, informing intervention, and interacting with complex physiological environments [1,2,3,4,5,6,7,8,9,10].

This shift has been driven by several converging developments. Firstly, advances in miniaturization have encouraged the design of more portable and clinically flexible support systems. Moreover, the progresses in decellularization, perfusion, organoid science, and bioprinting have expanded the possibility of reproducing structural and functional features of living tissues. Furthermore, digital medicine, simulation, and machine learning have widened the scope of artificial organs beyond replacement and toward prediction, personalization, and procedural optimization. Concurrently, the field continues to confront persistent and foundational obstacles, including hemocompatibility, long-term stability, immune acceptance, tissue vascularization, system integration, and translational scalability [1,3,6,7].

These tensions between promise and limitation define the present moment. The future of artificial organ engineering will depend not only on how well individual technologies perform in isolation, but on how effectively they can be combined into coherent therapeutic ecosystems. In this sense, this Special Issue offers a timely and meaningful portrait of the field. The ten contributions collected herein do not represent a single technological trajectory; instead, they reveal a broader intellectual movement toward smaller, smarter, and more biologically informed artificial organs that are more closely aligned with the complexity of human disease and patient-specific care [1,2,3,4,5,6,7,8,9,10].

The central direction highlighted in this Special Issue is the effort to move from hospital-bound organ support toward portable and potentially wearable therapeutic systems. Boscarino et al. explored the feasibility of an ultra-compact regenerative liver dialysis device, using validated mathematical models based on in vitro data to assess bilirubin removal by albumin-functionalized silica microspheres [1]. Their work is significant because it addresses one of the major unmet needs in extracorporeal organ support—namely, the possibility of reducing size and complexity without abandoning detoxification performance. Although the proposed system remains preliminary and distant from clinical use, the study points toward a future in which liver support may become more accessible, decentralized, and compatible with patient mobility [1]. In a related but broader tissue-engineering context, Zhu et al. introduced a portable perfusion and incubation platform capable of maintaining continuous flow under standard culture conditions while preserving tissue viability and enhancing spatial organization within engineered constructs [2]. Their system suggests that portability is not only a goal for artificial organ replacement devices, but also for the enabling bioreactor technologies that will underpin tissue maturation, transport, and distributed manufacturing in regenerative medicine [2].

The second major advancement concerns the recreation of complex tissue architecture through scaffold preservation, matrix engineering, and controlled biological microenvironments. McCarthy et al. established a robust protocol for the decellularization of human digits from long-term freezer storage and showed that cellular material could be effectively removed while preserving extracellular matrix composition, vascular integrity, and tendon mechanics [3]. This work is especially relevant because it extends decellularization research into vascularized composite allotransplantation and opens the possibility of off-the-shelf composite grafts for reconstructive surgery [3]. The study is also emblematic of a larger transition within the field—namely, the shift from simplified scaffold models toward anatomically and functionally complex biological templates. Halper offered a broad and thoughtful review of bioprinting that further illuminates this transition [4], which shows that bioprinting has progressed far beyond the fabrication of isolated structures and is now contributing to tissue regeneration, medical instrumentation, organoid development, and the emergence of four-dimensional constructs capable of dynamic change over time [4]. Taken together, these contributions suggest that the future artificial organ will likely be defined not only by function, but by its ability to reproduce spatial hierarchy, biological responsiveness, and integration with host tissues.

The third direction emerging from this Special Issue is the growing importance of physiologically relevant in vitro models as platforms for both disease understanding and artificial organ development. Song et al. demonstrated that a three-dimensional collagen matrix can reproduce key aspects of the desmoplastic stroma of pancreatic ductal adenocarcinoma and can actively promote tumor proliferation, migration, and glycolytic adaptation [5]. Their work reinforces a principle that is becoming increasingly central in bioengineering—namely, that tissue models must account for the physical and mechanical determinants of disease, not only the cellular ones [5]. Furthermore, Xu et al. extended this logic to toxicology and environmental health, proposing organoid models as a more human-relevant strategy for assessing the risks associated with per- and polyfluoroalkyl substances [6]. Their perspective is important because it expands the relevance of artificial tissue systems beyond replacement medicine and into predictive, preventive, and mechanistic applications [6]. Both studies remind us that the engineering of artificial organs cannot be separated from the engineering of disease models. The ability to replicate pathological microenvironments with sufficient fidelity will be indispensable for designing better devices, testing new materials, and anticipating patient-specific responses.

The interface between engineering innovation and procedural medicine constitutes another important theme of this Special Issue. Liu et al. developed and validated a three-dimensional printing-based simulator for transcatheter pulmonary valve replacement in complex native right ventricular outflow tracts [7]. Their findings showed reductions in crossing time, fluoroscopy time, and total operative time after simulation-based preparation, thereby supporting the practical value of patient-specific rehearsal in cardiovascular intervention [7]. This contribution highlights an often underappreciated truth: artificial organ engineering is not limited to implantable or extracorporeal devices, but includes the design of training environments, planning tools, and translational platforms that improve how therapies are delivered as well. In a different but equally relevant clinical setting, Le Picault et al. reported two successful thrombectomies in patients with a SynCardia total artificial heart who developed ischemic stroke while awaiting transplantation [8]. Although based on a small number of cases, this report is notable because it demonstrates that even highly complex recipients of artificial circulatory support can benefit from timely and standardized neurointerventional care [8]. The study reinforces the notion that the future of artificial organs will depend on multidisciplinary systems of care as much as on device design itself.

No vision of artificial organ engineering can ignore the problem of blood compatibility, which remains one of the most critical barriers to long-term success in blood-contacting devices. Kuchinka et al. provided a comprehensive review of coagulation, platelet activation, complement activation, and the surface engineering strategies developed to improve hemocompatibility [9]. Their analysis makes it clear that every blood-contacting artificial organ exists at a biologically sensitive boundary where thrombosis, inflammation, and material response converge [9]. This remains true across cardiovascular implants, extracorporeal support systems, dialysis membranes, and hybrid bioartificial platforms. The continued refinement of hemocompatible surfaces is therefore not a peripheral issue, but one of the enabling conditions for durable artificial organ function. In the coming years, progress in this area will likely determine which promising prototypes can become safe long-term clinical technologies.

The growing contribution of artificial intelligence and predictive modeling is represented in this Special Issue by the work of De Paoli et al., who compared offline and online learning strategies for blood glucose forecasting in subjects with type 1 diabetes during physical activity [10]. Their study is particularly instructive because it combines technical rigor with clinical realism and shows that, although adaptive online methods may seem conceptually attractive in dynamic physiological contexts, their increased computational burden did not translate into a decisive practical advantage over offline training in this setting [10]. This is an important lesson for the broader field. Intelligence in artificial organ engineering must not be judged by sophistication alone; instead, its value lies in interpretability, reliability, and clinically meaningful benefit. As predictive and control algorithms become more integrated into artificial organ systems, careful evaluation of their real-world utility will become increasingly necessary.

Taken together, the articles in this Special Issue address several critical knowledge gaps. They confront the challenge of portability in organ support and tissue culture [1,2]. They improve the biological realism of engineered tissues and disease models [3,4,5,6]. They strengthen the clinical bridge between device development and procedural practice [7,8]. They refine our understanding of blood–material interactions [9]. They also clarify both the possibilities and the current limits of computational forecasting in personalized medicine [10]. What emerges is not simply a collection of separate advances, but a more general redefinition of the field itself.

The next breakthroughs are unlikely to come from isolated innovation within a single domain; instead, it is more likely that they will arise at the intersections among scaffold biology and machine intelligence, among microphysiological systems and portable hardware, among patient-specific imaging and additive manufacturing, and among hemocompatible materials and adaptive sensing. The artificial organ of the future may no longer be best described as a device in the conventional sense, but may instead resemble an integrated therapeutic platform that senses, responds, remodels, predicts, and evolves with the patient.

Several future directions deserve particular attention. Firstly, the field must continue pursuing biologically complex constructs that incorporate vascular, immune, neural, and stromal elements with greater fidelity. The era of structurally simplified models is giving way to one in which physiological relevance determines translational value [3,4,5,6]. Secondly, portability and decentralization should become strategic objectives in both extracorporeal support and tissue engineering infrastructure. Devices and platforms able to function beyond highly specialized settings may reshape accessibility and continuity of care [1,2]. Thirdly, patient-specific digital pipelines should be further developed so that imaging, simulation, prediction, and fabrication can inform one another in real time [4,7,10]. Fourthly, durable blood compatibility must remain a priority, especially as the ambition of long-term implantable and extracorporeal systems continues to grow [9]. Finally, the translational pathway itself must be reimagined. Manufacturing reproducibility, regulatory science, validation across heterogeneous populations, and integration into clinical workflows will be as decisive as the underlying scientific discoveries.

In conclusion, the present Special Issue reflects a field rapidly expanding in scope while deepening in sophistication. Artificial organ engineering is no longer defined solely by the replacement of what has failed, but is, instead, increasingly concerned with preserving, anticipating, guiding, and regenerating. The contributions gathered herein show that the field is progressing toward technologies that are more compact, more intelligent, more biomimetic, and more clinically attuned. Additionally, they remind us that the most important advances still lie ahead—the real frontier is not only engineering a functional artificial organ, but engineering one that belongs meaningfully within the biological, clinical, and human realities it is meant to serve.
